# Long-term dynamics of density dependence reveals a more stable effect of the neighborhood on tree growth than tree survival

**DOI:** 10.1371/journal.pone.0316084

**Published:** 2025-01-22

**Authors:** Liping Wang, Junjie Wu, Fengxian Chen

**Affiliations:** College of Agriculture and Biological Science, Dali University, Dali, China; Mediterranean Institute for Advanced Studies, SPAIN

## Abstract

Density dependence is a vital mechanism for explaining tree species diversity. Empirical studies worldwide have demonstrated that neighbor density influences plant survival and growth in various communities. However, it remains unclear how neighbor density affects plant survival and growth over extended periods. We used data from tree censuses collected every five years from 1981 to 2015 (seven intervals) to assess how density dependence affects adult tree survival and growth by using generalized linear mixed models and the coefficients of variation in Barro Colorado Island plot. Linear regression models were used to assess whether the effects of density dependence on tree survival and growth correlated with species abundance. The results indicated that the effects of tree size (DBH) on tree survival and growth differed across all intervals. We found that the effects of heterospecific neighbor density on tree survival varied over time and consistently had significant negative impacts on tree growth. Conspecific neighbor densities had significant negative effects on tree survival and growth across all intervals. The effect of density dependence on tree growth was more stable than its impact on tree survival. Additionally, the relationship of species abundance and the effect of negative conspecific and heterospecific neighbor densities significantly affected tree growth but not survival, with negative and positive correlations to tree abundance over time, respectively. Our results revealed that neighboring density dependence can predict more accurately of tree growth than survival.

## Introduction

Density dependence mainly focuses on the interaction between a focal individual and its neighbors as the neighbor population increases over time. Conspecific negative density dependence (CNDD) [1,2] is an important mechanism driving the dynamics of plant communities [3,4] and has been proposed to explain the higher species diversity in some communities [4–6]. An increasing number of researches have revealed that the existence of CNDD is prevalent in plant communities [7–9].

CNDD reduces tree survival and growth when higher densities of conspecific neighbors surround them, creating opportunities for the establishment of other tree species [4,10]. Variability in CNDD is essential for explaining species diversity [3]; lower variability in CNDD strength leads to increased species richness and decreased variance in the relative species abundance [3]. CNDD varies according to abiotic (i.e., spatial, temporal, ontogenetic) and biotic (i.e., interspecific differences) factors. Although the importance of this mechanism is well-documented [11,12], its variation over time remains less understood.

Heterospecific density dependence can have both negative [13–15] and positive [14,16–19] effects on focal species survival. The positive effects of heterospecific neighbors may result from the species herd protection hypothesis [20,21]. The survival of target trees can be higher when surrounded by a higher number of heterospecific neighbors, called “heterospecific positive density dependence” (HPDD). Heterospecific neighbors can hinder the spread of host-specific pests only when the benefits of herd protection are greater than the disadvantages of resource competition, generalist pest propagation, or both, “species herd protection” is evident [21], as heterospecific neighbors can hinder the spread of host-specific natural enemies [21]. However, heterospecific neighborhoods negatively influenced tree survival or growth in most studies (i.e., heterospecific negative density dependence, HNDD). The effect of HNDD may be due to the interspecific competition for resources [22,23], similar to that of CNDD. Previous studies have shown that conspecific and heterospecific neighbors influenced tree survival or growth. However, it is still unknown whether the long-term effects of neighbors on tree growth are more or less stable than those on tree survival.

Long-term fluctuations in density dependence can alter community assembly and neighbor interactions. The strength of the density dependence may vary substantially over time, particularly across different environments [24]. Studies have investigated the effects of density dependence on seedlings and adults through temporal experiments [10,12,17,25]. However, these effects are often limited to the experiment’s duration [26–28], with limited information from long-term monitoring. Large shifts in density dependence over the years might result in inconsistent effects on population growth, making seedling dynamics appear loosely coupled with population dynamics and diversity [14].

Previous studies have found that species abundance can affect variation in density dependence among species [3,4,29]. LaManna et al. [4] reported that species abundance decreases with the strength of CNDD, and rare species suffer stronger CNDD at the global scale, maintaining a lower relative abundance [7,8,30]. Meanwhile, a stronger CNDD for common species provides space for rare species [31], leading to a community compensatory trend (CCT) [32,33] that promotes stable species coexistence in the community.

While the relationship between species abundance and the strength of density dependence is understood, its long-term effects are unclear and challenging to predict. Most studies on the effects of density dependence have focused on the dynamics of seedling survival and growth rates (e.g., [10,19,24,27,34–36]. In particular, relatively little is known about the effects of neighbors on adult tree survival and growth over time. Thus, disentangling the influence of density dependence remains challenging because the related mechanisms vary across time and space [23,37]. Long-term tree survival and growth tracking are essential for detecting forest succession processes and density-dependent trends.

In this study, we aimed to understand the effect of neighbors on tree survival and growth over time in a tropical rainforest and the relationship between species abundance and density dependence. Using long-term data from a Barro Colorado Island plot, we fitted generalized linear mixed models (GLMMs) and linear regression models. This study will enhance our understanding of diversity maintenance in species-rich communities. Specifically, we aim to address the following questions:

(1) What is the variability in the density dependence of tree survival and growth over time? (2) Are there significant differences in the effects of density dependence on tree survival and growth? and (3) Is there a relationship between species abundance and density dependence on tree survival and growth over time?

## Materials and methods

### Study site

This study was conducted in a 50-ha lowland tropical moist forest of Barro Colorado Island (BCI), Panama (9°10′ N, 79°51′ W). BCI plot was established to monitor the dynamics of woody adult trees ≥1 cm diameter at 1.3 m above-ground (DBH) between 1980 and 1982, and these trees were mapped, measured DBH, and identified to species [38–41]. Since 1985, the adult tree census was conducted each five years. The terrain is relatively flat, with an elevation range between 120 m and 160 m. A year-round mean temperature is 25.9°C, and annual precipitation averages 2,600 mm [42]. We used eight census data from 1980 to 2015, and 335332 trees have been measured (S1 Table). More detailed description of the 50-ha permanent plot can be found in Umaña et al. [42].

### Adult tree survival and growth rate

Each tree during the focal period was recorded as either alive (1) or dead (0). The difference in tree DBH between consecutive censuses and the time difference between these censuses were used to calculate the relative annual growth rate (RGR) for each tree. The RGR was calculated as (log (DBH_*t+1*_)—log (DBH_*t*_))/time [43], where the size in census *t* corresponds to the DBH of trees.

### Neighborhood variables

We calculated the total adult neighbor density (BAtot) as the summed basal area (BA) of nearby adults, weighted by their distance to the focal tree [44], using the formula:

$$BAtot=\sum_{i}^{N}\frac{{BA}_{i}}{{Distance}_{i}}$$ (1)

where N is the number of adult neighbors. All trees in the BCI plots were included as neighbors when computing the TA. Conspecific (BAcon) and heterospecific (BAhet) adult neighbor densities were calculated similarly. To avoid edge effects, trees within 20 m of the edge of the plot were not considered focal trees in this analysis [45].

### Data analysisc

We analyzed all living trees separately in each census interval [40,41]. Living trees in any census included survivors from the previous census and new recruits from the most recent census interval.

GLMMs were constructed using the lme4 package [46] in R software (v. 4.4.0) to model the probability of tree survival (with logit link and binomial errors) [47] and tree growth (RGR) (with identity link and Gaussian error) as functions of explanatory variables, assuming that tree survival and growth depend on tree DBH and the density of conspecific (BAcon) and heterospecific (BAhet) adult neighbors. In case of strong deviations from normality or homoscedasticity, all continuous explanatory variables were log-transformed. All continuous variables were standardized by subtracting the mean value of the variable (across all individuals in the analysis) and dividing it by one standard deviation before the analyses, which allowed us to directly compare the relative importance of these explanatory variables [48]. The means and ranges of all continuous explanatory variables used in the analysis are listed in S2 Table. Mean and range of growth rates and the number and proportion of dead trees in each census.

We divided the 50-ha (1000 m × 500 m) plot into 1250 (20 m × 20 m) subplots and assigned each tree to the quadrat number where it was located. To account for potential spatial autocorrelation between tree survival and growth due to unexplored habitats and other factors, we added random tree quadrat effects to our models. Previous studies suggested that this approach is sufficient to address autocorrelation [25,49]. Furthermore, we included species identity as a random effect because trees of different species are expected to respond differently to local neighborhood variables [24]. We also added species-specific random slopes for tree size (DBH), BAcon, and BAhet, as there were different relationships between these factors and tree survival and growth among the species.

The model parameters were estimated using maximum likelihood. The estimated coefficients represent the relative strength of the variables’ effects; coefficients > 0 indicate positive effects on tree survival or growth, whereas coefficients < 0 indicate negative effects. The coefficients of variation (CV) of all the estimated coefficients for all intervals were calculated. We also tested whether the effects of tree DBH, BAcon, and BAhet varied between tree survival and growth. We performed the Shapiro-Wilk test to test if the data were normally distributed and Bartlett’s test to test if the variances in each group were the same. We then used t-tests to determine whether the coefficient values of the three parameters showed significant differences between tree survival and tree growth.

To determine whether and how species abundance affects the detectability of density dependence among species, we added species-specific random slopes for conspecific and heterospecific adult neighbor densities to the models for the seven census intervals. The corresponding slopes show interspecific variations in response to the relevant variable. We then used linear regression models to test whether the conspecific adult density-dependent effects and heterospecific adult density-dependent effects of all species on tree survival and growth were correlated with species abundance. We also tested whether conspecific adult density-dependent and heterospecific adult density-dependent effects vary between tree survival and growth. We performed the Shapiro-Wilk test to test if the data were normally distributed and Bartlett’s test to test if the variances in each group were the same. We then used t-tests to determine whether the coefficient values of the two density parameters were significantly different for tree survival and growth.

All analyses were performed in R version 4.4.0 [50].

## Results

### Effects of density dependence on tree survival and growth

We constructed models to predict tree survival and growth, and the results showed that the proportion of fixed effects and total effects (fixed and random) on tree growth explained higher than on survival in seven intervals (S3 Table). Across all seven census intervals, the variance in the coefficients of focal tree size and conspecific and heterospecific adult neighbors on tree survival was larger than that on tree growth (Table 1). Tree size had a strong positive effect on tree survival, but the opposite was true for tree growth. Densities of conspecific neighbors (BAcon) showed significant negative effects on tree survival and growth at the seven intervals. The effects of the density of heterospecific neighbors (BAhet) on tree survival varied between intervals. BAhet had negative effects on tree survival in the second, fourth, fifth, and sixth intervals, whereas tree survival was positively correlated with BAhet in the seventh interval. There was no significant effect of BAhet on tree survival in the first and the third intervals. BAhet had significant negative effects on tree growth at the seven intervals. The variance in the coefficients (CV) of all variables for tree survival was larger than that for tree growth.

**Table 1:** The estimated coefficients of tree size, total basal area of conspecific, and heterospecific tree neighbors on tree survivals and growths for seven census intervals.

Interval	Survival			Growth		
	*DBH*	*BAcon*	*BAhet*	*DBH*	*BAcon*	*BAhet*
1	0.122***	–0.066***	0.007NS	–0.012***	–0.001***	–0.002***
2	0.144***	–0.107***	–0.045***	–0.030***	–0.002***	–0.005***
3	0.192***	–0.090***	–0.017NS	–0.025***	–0.001*	–0.005***
4	0.199***	–0.059***	–0.032*	–0.026***	–0.002*	–0.006***
5	0.086**	–0.050**	–0.048***	–0.023***	–0.002**	–0.004***
6	0.087**	–0.074***	–0.061***	–0.028***	–0.002***	–0.006***
7	0.309***	–0.047***	0.030**	–0.015***	–0.001***	–0.003***
CV	0.484	–0.309	–1.375	–0.296	–0.341	–0.341

Note: *DBH*, tree size; *BAcon*, total basal area of conspecific trees; *BAhet*, total basal area of heterospecific trees

*P < 0.05

**P < 0.01

***P < 0.001; NS, not significant. The coefficients of variation (CV) of all the estimated coefficients for all intervals were calculated.

### The variance of CNDD and HNDD along time

**Fig 1:**
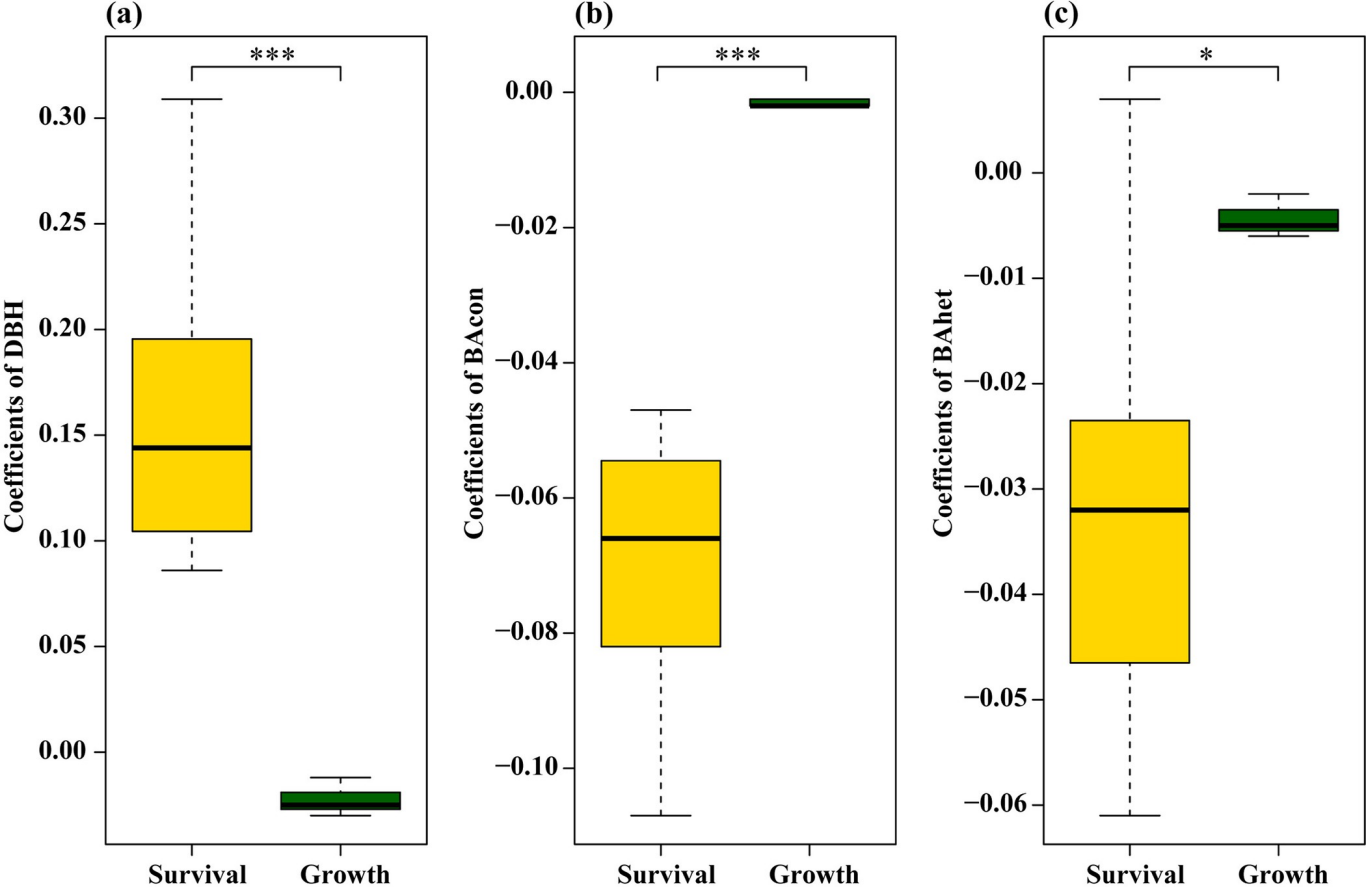
Estimates of DBH, BAcon (total basal area of conspecific trees), and BAhet (total basal area of heterospecific trees) for survival (gold) and growth (dark green). Error bars represent stand error (*P < 0.05; **P < 0.01; ***P < 0.001).

The effects of DBH on tree survival and growth differed significantly (*t* = 6.208, *P* < 0.001; Fig 1A). There were stronger negative effects of BAcon and BAhet on tree survival than on tree growth (*t* = –8.357, *P* < 0.001; *t* = –3.280, *P* = 0.017; respectively; Fig 1B and 1C). We found a larger variance in density-dependent effects on tree survival over time but a small variance in density-dependent effects on tree growth among intervals (Table 1; Fig 1).

### The relationship of species abundance and CNDD and HNDD

Linear regression models were used to test whether the specific random effects on BAcon and BAhet (i.e., the random slopes for each species in the models for tree survival and growth) correlated with species abundance. The effects of BAcon on tree survival were not significantly correlated with species abundance in the first, third, fourth, and seventh intervals but were significantly correlated in the second, fifth, and sixth intervals. The effects of BAhet on tree survival were not significantly correlated with species abundance in the first and third intervals. Both the effects of BAcon and BAhet on tree growth were significantly correlated with species abundance (*P* < 0.01) (Table 2). Negative relationships were observed between species abundance and the coefficients of BAcon, indicating that high species abundance was accompanied by high CNDD. Positive relationships between species abundance and the coefficients of BAhet were found, suggesting that high species abundance leads to low strength of HNDD.

**Table 2:** The slope and *P*-values in linear regression models to test whether BAcon effects and BAhet effects of all species for survival and growth correlated with the species abundance in seven intervals.

Interval	Survival				Growth			
	*BAcon*		*BAhet*		*BAcon*		*BAhet*	
	Slope	*P*	Slope	*P*	Slope	*P*	Slope	*P*
1	–2.87×10^−6^	0.087	4.90×10^−7^	0.055	–2.64×10^−6^	**<0.001**	2.01×10^−6^	**<0.001**
2	–5.95×10^−6^	**<0.001**	1.15×10^−6^	**0.020**	–3.02×10^−6^	**<0.001**	4.01×10^−6^	**<0.001**
3	–1.20×10^−6^	0.341	1.39×10^−6^	0.145	–3.03×10^−6^	**<0.001**	4.22×10^−6^	**0.002**
4	–2.30×10^−6^	0.077	1.81×10^−6^	**0.013**	–3.54×10^−6^	**<0.001**	3.85×10^−6^	**<0.001**
5	–2.42×10^−6^	**0.048**	2.14×10^−6^	**<0.001**	–4.40×10^−6^	**<0.001**	2.62×10^−6^	**0.001**
6	–3.51×10^−6^	**0.015**	3.53×10^−6^	**<0.001**	–2.61×10^−6^	**0.002**	3.86×10^−6^	**0.002**
7	1.39×10^−6^	0.076	1.08×10^−6^	**0.038**	–3.43×10^−6^	**<0.001**	2.36×10^−6^	**<0.001**

Note: The statistically significant results (*P<* 0.05) are in bold font.

## Discussion

Density dependence can help drive tree dynamics in tropical forest communities [51]. In this study, we examined whether the strength of the density-dependent effects on tree survival and growth varied over time. Our results showed that the variance in the coefficients of all variables for tree survival was larger than that for tree growth. Furthermore, we found that the strengths of CNDD and HNDD in tree growth were strongly related to species abundance over time, whereas the relationship between the strengths of CNDD and HNDD in tree survival and species abundance was uncertain over time.

### Tree size and density dependence on tree survival and growth over time

Tree size (DBH) was one of the most important predictors of tree survival and growth [51]. We found that DBH positively affected tree survival in the BCI forest. This result is consistent with previous studies that showed that trees had higher survival rates as they grew larger [23,52–54]. However, tree size had negative effects on tree growth, similar to large trees exhibiting significantly higher survival probabilities and lower growth rates [53].

The effects of CNDD are common in plant communities [12], and they drive forest diversity [4]. We found that the effects of BAcon showed consistent and significant negative effects on tree survival and growth. Previous studies have evidenced that CNDD effect is usually caused by host-specific natural enemies [55] and soil pathogens [56]. However, the effect of BAhet on tree survival changed from negative to positive over time, which suggested that heterospecific neighbor effects transformed from HNDD to HPDD. Tree survival and growth decreased with the increasing heterospecific tree density, probably due to competition for resources [57]. The positive effect of BAhet on tree survival in our study is similar to that reported in studies conducted in the BCI [14] and in a temperate forest in northeastern China [15]. This likely resulted from fewer encounters between the host and its specialized natural enemies due to increased heterospecific crowding [21,29]. In addition, this positive relationship may due to the fact that trees survive well in the suitable habitat and live at a higher density. The coefficients of BAcon and BAhet for tree survival showed significant fluctuations among intervals. However, the intensities of CNDD and HNDD for tree growth remained stable within a small range over time. It is important to note that our intervals included the El Niño-Southern Oscillation event of 1985–1990. In this interval, the negative effect of conspecific density dependence was the strongest for tree survival. Variations in environmental conditions over time may lead to uncertainty regarding the effects of CNDD on tree survival [58,59]. Habitat heterogeneity and climate change play indispensable roles in the survival and growth of trees, although these factors were not considered in the present study.

At the same site, Weng et al. [60] found that the effect of neighborhood interaction on growth increased over time. Zhang et al. [61] indicated that competition was the primary factor causing long-term changes in tree mortality, growth, and recruitment. To test whether CNDD varies with forest age, Johnson et al. [8] reanalyzed data from different successional forests and found that the pattern of CNDD was consistent between different age classes, indicating that CNDD is not contingent on successional dynamics. The strength of density dependence varied over time and was influenced by water availability and temperature in a tropical rainforest [62]. Density-dependent processes fluctuated over 50 years in an ecotone forest in the southwestern Canadian boreal forest [63], and the strength and direction of these demographic and spatial processes varied in response to time and disturbance severity. Stump and Comita [64] stated that interspecific variation in CNDD can make species less likely to coexist and not temporal variation in negative dependence, which makes communities less stable. A host of studies found considerable differences of density dependence in the effects of different factors among ecological guilds and species, such as shade-tolerant guilds, tree-size guilds, lifer-history guilds and species level [14,23].

The effective survival and growth of adult trees and density dependence are crucial for the successful regeneration of trees, and they represent vital regulators in the maintenance and structuring of hyper-diverse plant communities [65]. The significant effects of heterospecific neighbors on tree growth was more stable than survival when the predictions of density dependence were tested in this plot. The uncertainty of neighborhood effects on tree survival increases the complexity of the research. Additionally, the explanatory powers of models on tree growth were stronger (S3 Table). Therefore, the sensitivity of tree growth is more stable to neighborhood effects over time. These findings also highlight the importance of creating and regularly surveying long-term monitoring plots [63].

### The relationship between the strength of density dependence and species abundance

Variations in the strength of the CNDD among forests have also been linked to the relative abundance [43]. In our study, the relationship between the effects of BAcon and BAhet on tree growth and species abundance was similar for all species, whereas the relationship between the effects of BAcon and BAhet on tree survival and species abundance was not. Our results showed that species abundance increased with the strength of CNDD on tree survival and growth and decreased the negative effect of BAhet. This result is in accordance with several studies [3,8,66]; common species suffer stronger CNDD, and rare species suffer weak CNDD. A greater abundance increases the frequency of conspecific interactions, leading to the high mortality associated with CNDD [67]. Additionally, Lebrija-Trejos et al. [68] observed a negative relationship between seedling CNDD and species abundance in a BCI plot.

Our results show a positive relationship between species abundance and heterospecific density dependence on tree survival and growth. This was consistent with the findings of John et al. [69], who illustrated that the heterospecific basal area and heterospecific abundance were strongly positively correlated with the recruitment of *Lagerstroemia*. The effect of HPDD may be because a large number of heterospecific neighbors could decrease the probability of encounters with its host specificity and pathogens, which facilitates tree survival and growth [21]. Moreover, it could also cause niche partitioning among species to reduce competitive exclusion compared with conspecifics [70].

In contrast, Comita et al. [7] found that seedlings of more abundant species suffered less from conspecific neighbor effects than rare species in the community in BCI and suggested that local-scale density dependence constrained the abundance of a species, and these constraints varied among species. They concluded that previous studies probably underestimated both the variation and mean strength of density dependence in plant communities. Moreover, this phenomenon may be due to the strong effects of soil pathogens on rare species [30,71].

There was no significant relationship between the density dependence effect on tree survival and species abundance, which increases the complexity of the related analysis. We speculated that the effect of density dependence on species abundance may be related to the surrounding environment [72], although we did not include environmental factors in this analysis. In summary, species abundance is closely related to growth-based density dependence.

## Conclusions

In this study, we compared the effects of tree size and density dependence on tree survival and growth over time, and the relationship between species abundance and the strength of density dependence on tree survival and growth. Overall, our research suggests that the relative significance of these factors varies dramatically among on tree survival and growth, and the sensitivity of tree growth, rather than survival, to the effects of neighbors is more stable. For future studies of density dependence, we should prioritize adult tree growth as response variable, which would greatly improve the accuracy and stability of the neighborhood effect.

## Supporting information

S1 Table Adult trees of the 50-ha BCI plot along the eight censuses. (DOCX)

S2 Table The means and ranges of all continuous explanatory variables in Generalized linear mixed models (GLMMs). (DOCX)

S3 Table The predictive capacity for fixed effects (R^2^_*mar*_) and total (fixed and random) effects (R^2^_*con*_) values of models on tree survival and growth. (DOCX)
