# Supplementary material for: Long-term dynamics of density dependence reveals a more stable effect of the neighborhood on tree growth than tree survival
Source: PLoS One. 2025 Jan 22;20(1):e0316084. doi: 10.1371/journal.pone.0316084 (PMC11753649; doi:10.1371/journal.pone.0316084)
Supplement: S1 Table — (DOCX) [file pone.0316084.s001.docx]

**Supplemental Table 1. Adult trees of the 50-ha BCI plot along the eight censuses.**

| **Censuses** | **Number of trees** | **Number of trees** **alive** | **Number of species** |
| --- | --- | --- | --- |
| 1st | 263934 | 263896 | 307 |
| 2nd | 299447 | 272520 | 309 |
| 3rd | 331053 | 289329 | 315 |
| 4th | 318419 | 268513 | 311 |
| 5th | 299447 | 250176 | 311 |
| 6th | 288477 | 245776 | 311 |
| 7th | 318187 | 247859 | 313 |
| 8th | 335332 | 248836 | 311 |
