# Supplementary material for: Long-term dynamics of density dependence reveals a more stable effect of the neighborhood on tree growth than tree survival
Source: PLoS One. 2025 Jan 22;20(1):e0316084. doi: 10.1371/journal.pone.0316084 (PMC11753649; doi:10.1371/journal.pone.0316084)
Supplement: S2 Table — (DOCX) [file pone.0316084.s002.docx]

**Supplemental Table 2. The means and ranges of all continuous explanatory variables in Generalized linear mixed models (GLMMs).**

| **Interval** | ***DBH (cm)*** | | ***BAcon*** | | ***BAhet*** | | **Growth rate** | | **Dead** | |
| --- | --- | --- | --- | --- | --- | --- | --- | --- | --- | --- |
|  | ***Rang*** | ***Mean*** | ***Rang*** | ***Mean*** | ***Rang*** | 200044 | ***Rang*** | ***Mean*** | ***Number*** | ***Proportion (%)*** |
| 1 | 1.00~350.00 | 4.34 | 0.00~5.04×10^13^ | 2.51×10^8^ | 12092~2.25×10^8^ | 218838 | –0.83~0.61 | 0.02 | 68219 | 29.30 |
| 2 | 0.40~285.00 | 4.15 | 0.00~9.03×10^13^ | 4.53×10^8^ | 12509~1.79×10^8^ | 201317 | –0.70~0.84 | 0.05 | 40737 | 16.92 |
| 3 | 0.20~816.90 | 4.88 | 0.00~1.24×10^14^ | 6.15×10^8^ | 14765~1.30×10^8^ | 190563 | –0.74~0.87 | 0.02 | 36751 | 14.38 |
| 4 | 0.10~246.80 | 4.66 | 0.00~1.66×10^14^ | 8.24×10^8^ | 12157~7.63×10^7^ | 187551 | –0.69~0.93 | 0.02 | 36499 | 15.35 |
| 5 | 1.00~246.80 | 4.74 | 0.00~1.82×10^14^ | 9.71×10^8^ | 14253~1.02×10^8^ | 178109 | –0.76~0.80 | 0.02 | 31152 | 14.05 |
| 6 | 1.00~276.50 | 4.82 | 0.00~1.89×10^14^ | 1.12×10^9^ | 15615~7.58×10^7^ | 1.32×10^5^ | –0.69~0.99 | 0.03 | 29977 | 13.78 |
| 7 | 1.00~247.70 | 4.76 | 0.00~1.86×10^14^ | 1.21×10^9^ | 14050~7.35×10^12^ | 7.97×10^7^ | –0.18~0.56 | 0.02 | 41656 | 18.95 |

Note: *DBH*, tree size; *BAcon*, conspecific density; *BAhet*, heterospecific density. The minimum DBH of the 2–4 intervals was < 1 cm, owing to the severed heads of trees in these intervals.
