# Supplementary material for: Long-term dynamics of density dependence reveals a more stable effect of the neighborhood on tree growth than tree survival
Source: PLoS One. 2025 Jan 22;20(1):e0316084. doi: 10.1371/journal.pone.0316084 (PMC11753649; doi:10.1371/journal.pone.0316084)
Supplement: S3 Table — (DOCX) [file pone.0316084.s003.docx]

**Supplemental Table 3. The predictive capacity for fixed effects (*R^2^_mar_*) and total (fixed and random) effects (*R^2^_con_*) values of models on tree survival and growth.**

| **Interval** | **Survival** | | **Growth** | |
| --- | --- | --- | --- | --- |
|  | ***R^2^_mar_*** | ***R^2^_con_*** | ***R^2^_mar_*** | ***R^2^_con_*** |
| 1 | 0.002 | 0.204 | 0.071 | 0.411 |
| 2 | 0.007 | 0.339 | 0.201 | 0.635 |
| 3 | 0.009 | 0.385 | 0.094 | 0.757 |
| 4 | 0.009 | 0.335 | 0.100 | 0.778 |
| 5 | 0.002 | 0.364 | 0.120 | 0.637 |
| 6 | 0.004 | 0.342 | 0.134 | 0.715 |
| 7 | 0.020 | 0.305 | 0.101 | 0.634 |
